# Crystal structure of 2-(4-acetyl­anilino)-2-oxoethyl 3-(4-hy­droxy­phen­yl)propionate

**DOI:** 10.1107/S205698901600894X

**Published:** 2016-06-10

**Authors:** Zaman Ashraf, Daeyoung Kim, Sung-Yum Seo, Sung Kwon Kang

**Affiliations:** a Department of Biology, College of Natural Sciences, Kongju National University, Gongju 314-701, Republic of Korea; b Department of Chemistry, Allama Iqbal Open University, Islamabad 44000, Pakistan; c Department of Chemistry, Chungnam National University, Daejeon 305-764, Republic of Korea

**Keywords:** crystal structure, cinnamate ester, N—H⋯O and O—H⋯O hydrogen bonds, tyrosinase inhibitor

## Abstract

In the title compound, C_19_H_19_NO_5_, the amide carbonyl O atom is positioned *anti* to the other two carbonyl O atoms. The 4-hy­droxy­hydro­cinnamate fragment is disordered over two positions with an occupancy ratio of 0.729 (12):0.271 (12). The N—(C=O)—C plane of the acetamide group and the acetate O—(C=O)—C plane are almost co-planar; the acetamide plane makes dihedral angles of 1.9 (6) and 16.0 (19)°, respectively, with the acetate planes of the major and minor occupancy components. In the crystal, N—H⋯O, O—H⋯O and C—H⋯O hydrogen bonds link the mol­ecules into a supra­molecular sheet structure parallel to (102).

## Abstract 2

In the crystal of the title compound, a supra­molecular sheet structure is formed through N—H⋯O, O—H⋯O and C—H⋯O hydrogen bonds.

## Chemical context   

**Figure 1:**
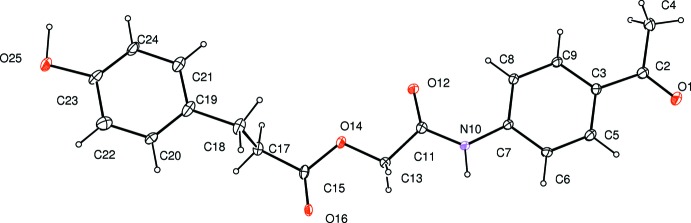
The mol­ecular structure of the title compound, showing the atom-numbering scheme and 30% probability ellipsoids. Only the major occupancy disorder component is shown.

Hy­droxy-substituted aromatic compounds with additional ester and amide functionalities have been reported to be potential tyrosinase inhibitors (Miliovsky *et al.*, 2013 ▸; Takahashi & Miyazawa, 2011 ▸). Tyrosinase is a key enzyme present in melanocytes, which is involved in the biosynthesis of melanin. The abnormal production and accumulation of melanin causes a number of hyperpigmentation disorders such as freckles, melasma, lentigo senilis and pigmented acne scars (Lynde *et al.*, 2006 ▸; Cullen, 1998 ▸). Tyrosinase has also been linked to melanoma, a skin-cancer type that arises from the aberrant proliferation of melanocytes (Uong & Zon, 2010 ▸). It has also been reported that tyrosinase is one of the main causes of most fruit and vegetable damage during post-harvest handling and processing, leading to quicker degradation and shorter shelf life (Yi *et al.*, 2010 ▸). Therefore, the synthesis of safe and effective tyrosinase inhibitors is of great concern in the medical, agricultural and cosmetic industries. The synthesis and tyrosinase inhibitory activity of hy­droxy-substituted phenyl esters is currently an ongoing research topic in our lab (Ashraf *et al.*, 2015 ▸). In view of the tyrosinase inhibitory potential of hy­droxy-substituted aromatic compounds, the title compound (Fig. 1 ▸) has been synthesized and characterized by single crystal X-ray diffraction.
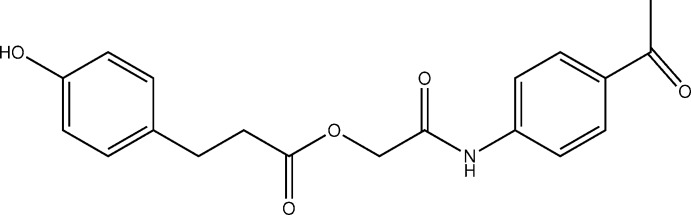


## Structural commentary   

The fragment O1/O12/N10/C2–C9/C11/C13 including the acetamide group is almost planar with an r.m.s. deviation of 0.034 (11) Å. The 4-hy­droxy­hydro­cinnamate fragment is disordered over two positions with occupancy ratio of 0.729 (12):0.271 (12). The acetamide plane O12/N10/C11/C12 makes dihedral angles of 1.9 (6) and 16.0 (19)°, respectively, with the disordered acetate planes O14/O16/C15/C17 and O14*A*/O16*A*/C15*A*/C17*A*. The carbonyl O1 and O16 atoms are positioned *anti* with respect to the carbonyl O12 atom. These C=O bond lengths are in the range 1.176 (12)–1.226 (6) Å.

## Supra­molecular features   

**Figure 2:**
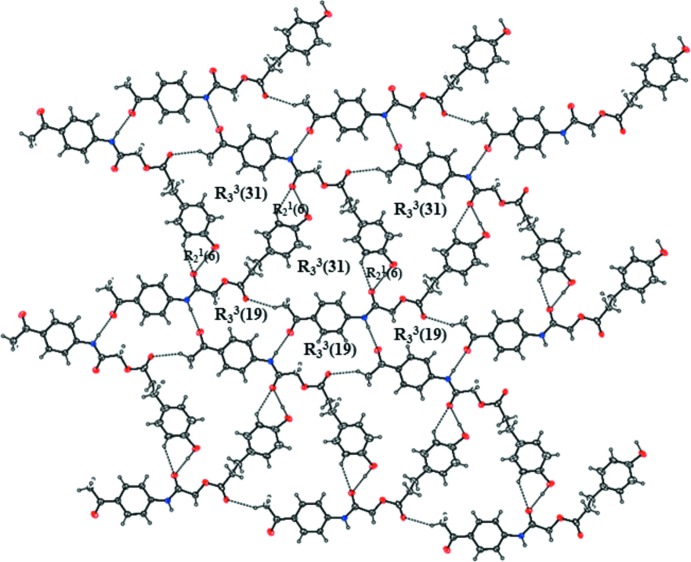
The sheet structure of mol­ecules linked by N—H⋯O, O—H⋯O, and C—H⋯O hydrogen bonds (dashed lines). Only the major occupancy disorder components are shown.

**Figure 3:**
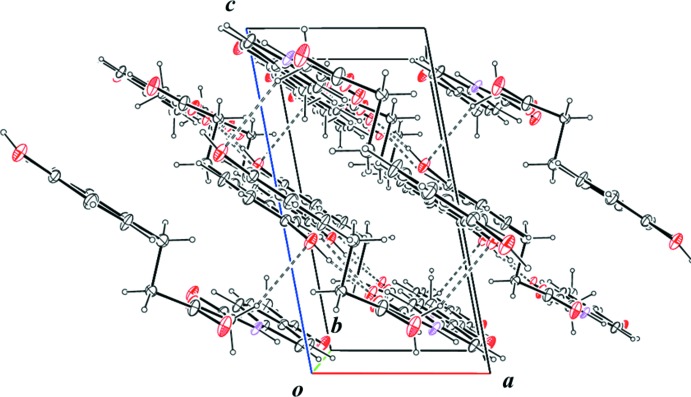
Part of the packing diagram of the title compound, showing the C—H⋯O hydrogen bonds (dashed lines) between the hydrogen-bonded sheets. Only the major disorder components are shown.

In the crystal, mol­ecules are linked *via* N—H⋯O, O—H⋯O and C—H⋯O hydrogen bonds (N10—H10⋯O1^i^, O25—H25⋯O12^ii^, C4—H4*A*⋯O16^iii^ and C24—H24⋯O12^ii^; Table 1 ▸), forming a sheet parallel to (102) (Fig. 2 ▸). In the sheet, these hydrogen bonds form 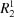
(6), 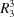
(19) and 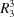
(31) graph-set motifs. There are also weak C—H⋯O hydrogen bonds (C13—H13*B*⋯O25^iv^ and C13—H13*B*⋯O25*A* ^iv^; Table 1 ▸) between the sheets (Fig. 3 ▸).

## Database survey   

A search of the Cambridge Structural Database (Version 5.37 with two updates, Groom *et al.*, 2016 ▸) returned three entries for crystal structures with ethyl hydro­cinnamate as the main skeleton (BESTIC: Böjthe-Horváth *et al.*, 1982 ▸; FUZYOQ: Wang *et al.*, 2015 ▸; NAXVIR: Hassan & Wang, 1997 ▸). There are 76 entries of organic compounds with the 4-acetyl­anilino group.

## Synthesis and crystallization   

**Figure 4:**
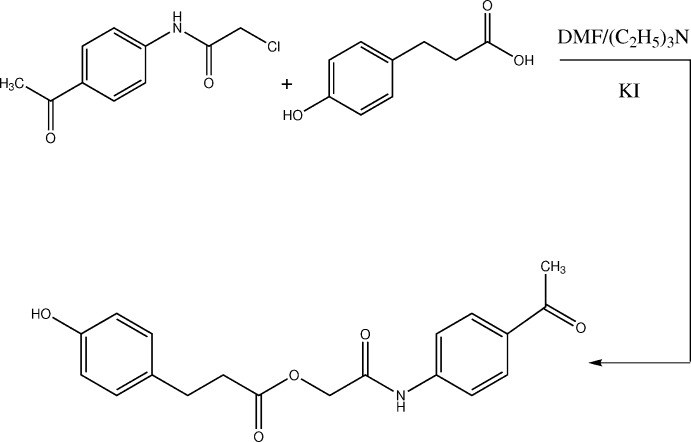
Reaction scheme for the synthesis of the title compound.

The title compound was synthesized by direct condensation of 4-hy­droxy­phenyl propanoic acid with *N*-(4-acetyl­phen­yl)-2-chloro­acetamide in the presence of dimethyl formamide (DMF) solvent and tri­ethyl­amine base (Fig. 4 ▸). The reaction mixture was stirred overnight at room temperature. Then the mixture was poured into finely crushed ice and extracted with ethyl acetate. It was washed with 5% HCl and 5% sodium hydroxide, and finally with aqueous NaCl solution. The organic layer was dried over anhydrous magnesium sulfate, filtered and the solvent was removed under reduced pressure to afford the crude product. The title compound was purified by silica gel column chromatography using ethyl acetate and *n*-hexane (3:1) as eluent. The single crystals were obtained from a solvent mixture of ethyl acetate/*n*-hexane (3:1) upon slow evaporation at room temperature (yield 78%, m.p. 419–421 K). FTIR ν_max_ cm^−1^: 3428 (N—H), 3354 (O—H), 2971 (*sp* ^2^ C—H), 2887 (*sp* ^3^ C—H), 1735 (C=O ester), 1646 (C=O amide), 1601 (C=C aromatic), 1154 (C—O, ester).

## Refinement   

Crystal data, data collection and structure refinement details are summarized in Table 2 ▸. The 4-hy­droxy­hydro­cinnamate fragment, O16/O25/C15–C24, was found to be disordered over two positions and the occupancy ratio was refined to 0.729 (12):0.271 (12). Atoms O16*A*, O25*A* and C15*A*–C24*A* of the minor component were refined isotropically. Planarity restraints were applied for atoms C18–C24, O25, C18*A*–C24*A* and O25*A*. Bond-distance restraints were also applied for C20, C22, C23, O16*A* and C15*A*–C24*A*. H10 and H25 of the NH and OH groups, respectively, were located in a difference Fourier map and the coordinates were refined with *U*_iso_(H) = 1.2*U*_eq_(N) and 1.5*U*_eq_(O) [N—H = 0.86 (5) Å and O—H = 0.95 (12) Å]. H25*A* of the minor occupancy OH group was refined with a restraint of O—H = 0.90 (2) Å, and with *U*_iso_(H) = 1.5*U*_eq_(O). All other H atoms were included as riding atoms, with C—H = 0.93–0.97 Å and with *U*_iso_(H) = 1.5*U*_eq_(C) for methyl H atoms or 1.2*U*_eq_(C) otherwise.

## Supplementary Material

Crystal structure: contains datablock(s) I. DOI: 10.1107/S205698901600894X/is5453sup1.cif

Structure factors: contains datablock(s) I. DOI: 10.1107/S205698901600894X/is5453Isup2.hkl

Supporting information file. DOI: 10.1107/S205698901600894X/is5453Isup3.cml

CCDC reference: 1483293

Additional supporting information: crystallographic information; 3D view; checkCIF report

## Figures and Tables

**Table 1:** Hydrogen-bond geometry (Å, °)

*D*—H⋯*A*	*D*—H	H⋯*A*	*D*⋯*A*	*D*—H⋯*A*
N10—H10⋯O1^i^	0.86 (5)	2.11 (5)	2.925 (5)	159 (4)
O25—H25⋯O12^ii^	0.95 (12)	1.93 (11)	2.87 (3)	172 (13)
C4—H4*A*⋯O16^iii^	0.96	2.59	3.416 (10)	144
C13—H13*B*⋯O25^iv^	0.97	2.60	3.458 (17)	147
C13—H13*B*⋯O25*A* ^iv^	0.97	2.50	3.34 (4)	145
C24—H24⋯O12^ii^	0.93	2.57	3.284 (11)	133

Symmetry codes: (i) 
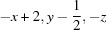
; (ii) 
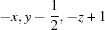
; (iii) 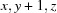; (iv) 
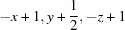
.

**Table 2:** Experimental details

Crystal data	
Chemical formula	C_19_H_19_NO_5_
*M*_r_	341.35
Crystal system, space group	Monoclinic, *P*2_1_
Temperature (K)	296
*a*, *b*, *c* (Å)	5.510 (3), 14.809 (9), 10.824 (7)
β (°)	100.757 (7)
*V* (Å^3^)	867.7 (9)
*Z*	2
Radiation type	Mo *K*α
μ (mm^−1^)	0.10
Crystal size (mm)	0.26 × 0.25 × 0.23

Data collection	
Diffractometer	Bruker SMART CCD area-detector
No. of measured, independent and observed [*I* > 2σ(*I*)] reflections	6909, 3403, 1798
*R*_int_	0.031
(sin θ/λ)_max_ (Å^−1^)	0.627

Refinement	
*R*[*F* ^2^ > 2σ(*F* ^2^)], *wR*(*F* ^2^), *S*	0.053, 0.126, 1.00
No. of reflections	3403
No. of parameters	276
No. of restraints	25
H-atom treatment	H atoms treated by a mixture of independent and constrained refinement
Δρ_max_, Δρ_min_ (e Å^−3^)	0.14, −0.17

Computer programs: *SMART* and *SAINT* (Bruker, 2012 ▸), *SHELXS2013* (Sheldrick, 2008 ▸), *SHELXL2013* (Sheldrick, 2015 ▸), *ORTEP-3 for Windows* (Farrugia, 2012 ▸) and *publCIF* (Westrip,2010 ▸).

## supplementary crystallographic information

### Crystal data

**Table:**

C_19_H_19_NO_5_	*F*(000) = 360
*M**_r_* = 341.35	*D*_x_ = 1.306 Mg m^−^^3^
Monoclinic, *P*2_1_	Mo *K*α radiation, λ = 0.71073 Å
*a* = 5.510 (3) Å	Cell parameters from 1529 reflections
*b* = 14.809 (9) Å	θ = 2.4–20.5°
*c* = 10.824 (7) Å	µ = 0.10 mm^−^^1^
β = 100.757 (7)°	*T* = 296 K
*V* = 867.7 (9) Å^3^	Block, colourless
*Z* = 2	0.26 × 0.25 × 0.23 mm

### Data collection

**Table:**

Bruker SMART CCD area-detector diffractometer	*R*_int_ = 0.031
Radiation source: fine-focus sealed tube	θ_max_ = 26.5°, θ_min_ = 1.9°
φ and ω scans	*h* = −6→6
6909 measured reflections	*k* = −18→18
3403 independent reflections	*l* = −13→13
1798 reflections with *I* > 2σ(*I*)	

### Refinement

**Table:**

Refinement on *F*^2^	25 restraints
Least-squares matrix: full	Hydrogen site location: mixed
*R*[*F*^2^ > 2σ(*F*^2^)] = 0.053	H atoms treated by a mixture of independent and constrained refinement
*wR*(*F*^2^) = 0.126	*w* = 1/[σ^2^(*F*_o_^2^) + (0.0507*P*)^2^ + 0.0787*P*] where *P* = (*F*_o_^2^ + 2*F*_c_^2^)/3
*S* = 1.00	(Δ/σ)_max_ < 0.001
3403 reflections	Δρ_max_ = 0.14 e Å^−^^3^
276 parameters	Δρ_min_ = −0.17 e Å^−^^3^

### Special details

**Table:**

Geometry. All esds (except the esd in the dihedral angle between two l.s. planes) are estimated using the full covariance matrix. The cell esds are taken into account individually in the estimation of esds in distances, angles and torsion angles; correlations between esds in cell parameters are only used when they are defined by crystal symmetry. An approximate (isotropic) treatment of cell esds is used for estimating esds involving l.s. planes.

### Fractional atomic coordinates and isotropic or equivalent isotropic displacement parameters (Å^2^)

**Table:**

	*x*	*y*	*z*	*U*_iso_*/*U*_eq_	Occ. (<1)
O1	1.0367 (8)	1.4286 (2)	0.0585 (4)	0.0831 (15)	
C2	0.8795 (10)	1.3999 (4)	0.1151 (5)	0.0551 (15)	
C3	0.8377 (10)	1.3024 (3)	0.1253 (5)	0.0451 (14)	
C4	0.7281 (11)	1.4662 (3)	0.1753 (6)	0.0689 (18)	
H4A	0.7711	1.5267	0.1559	0.103*	
H4B	0.7615	1.4578	0.2648	0.103*	
H4C	0.5557	1.4562	0.1434	0.103*	
C5	0.9784 (9)	1.2426 (4)	0.0675 (5)	0.0555 (16)	
H5	1.0986	1.2651	0.0258	0.067*	
C6	0.9408 (10)	1.1512 (3)	0.0717 (5)	0.0523 (15)	
H6	1.0375	1.1123	0.0339	0.063*	
C7	0.7613 (9)	1.1162 (3)	0.1315 (5)	0.0459 (14)	
C8	0.6218 (11)	1.1739 (3)	0.1902 (5)	0.0572 (17)	
H8	0.5016	1.1509	0.2315	0.069*	
C9	0.6618 (9)	1.2656 (3)	0.1872 (5)	0.0540 (16)	
H9	0.5684	1.3039	0.2278	0.065*	
N10	0.7347 (8)	1.0214 (3)	0.1292 (4)	0.0511 (13)	
H10	0.836 (9)	0.995 (4)	0.089 (4)	0.061*	
C11	0.5765 (10)	0.9683 (4)	0.1782 (5)	0.0500 (15)	
O12	0.4168 (7)	0.9955 (2)	0.2321 (4)	0.0621 (11)	
C13	0.6264 (11)	0.8699 (3)	0.1599 (5)	0.0509 (15)	
H13A	0.6134	0.8570	0.0710	0.061*	
H13B	0.7923	0.8548	0.2024	0.061*	
O14	0.4514 (7)	0.8175 (2)	0.2098 (4)	0.0650 (12)	
C15	0.4672 (17)	0.7263 (6)	0.1902 (11)	0.044 (3)	0.729 (12)
O16	0.631 (3)	0.6938 (5)	0.1497 (16)	0.074 (4)	0.729 (12)
C17	0.2874 (14)	0.6724 (5)	0.2509 (9)	0.048 (2)	0.729 (12)
H17A	0.2446	0.6169	0.2042	0.058*	0.729 (12)
H17B	0.1372	0.7070	0.2487	0.058*	0.729 (12)
C18	0.4021 (15)	0.6501 (7)	0.3868 (8)	0.070 (3)	0.729 (12)
H18A	0.5659	0.6254	0.3893	0.084*	0.729 (12)
H18B	0.4200	0.7054	0.4356	0.084*	0.729 (12)
C19	0.2509 (13)	0.5829 (7)	0.4479 (7)	0.063 (3)	0.729 (12)
C20	0.2881 (15)	0.4932 (7)	0.4377 (8)	0.065 (3)	0.729 (12)
H20	0.4061	0.4732	0.3928	0.078*	0.729 (12)
C21	0.0769 (17)	0.6103 (8)	0.5129 (10)	0.063 (3)	0.729 (12)
H21	0.0479	0.6717	0.5202	0.076*	0.729 (12)
C22	0.154 (2)	0.4300 (8)	0.4927 (10)	0.085 (3)	0.729 (12)
H22	0.1837	0.3686	0.4852	0.102*	0.729 (12)
C23	−0.024 (2)	0.4599 (14)	0.5590 (11)	0.065 (6)	0.729 (12)
C24	−0.059 (2)	0.5493 (11)	0.5684 (12)	0.060 (4)	0.729 (12)
H24	−0.1759	0.5704	0.6130	0.072*	0.729 (12)
O25	−0.156 (4)	0.389 (2)	0.6129 (17)	0.079 (6)	0.729 (12)
H25	−0.24 (2)	0.420 (8)	0.669 (14)	0.119*	0.729 (12)
C15A	0.526 (5)	0.7305 (17)	0.239 (2)	0.033 (7)*	0.271 (12)
O16A	0.647 (11)	0.698 (3)	0.164 (6)	0.13 (2)*	0.271 (12)
C17A	0.344 (5)	0.6930 (19)	0.317 (3)	0.055 (8)*	0.271 (12)
H17C	0.3841	0.7156	0.4029	0.066*	0.271 (12)
H17D	0.1770	0.7116	0.2813	0.066*	0.271 (12)
C18A	0.363 (4)	0.5920 (18)	0.317 (2)	0.075 (8)*	0.271 (12)
H18C	0.5348	0.5749	0.3420	0.090*	0.271 (12)
H18D	0.3055	0.5702	0.2321	0.090*	0.271 (12)
C19A	0.215 (3)	0.5467 (15)	0.4037 (16)	0.044 (7)*	0.271 (12)
C20A	0.256 (4)	0.4531 (14)	0.412 (2)	0.045*	0.271 (12)
H20A	0.3644	0.4259	0.3672	0.054*	0.271 (12)
C21A	0.054 (5)	0.5854 (17)	0.471 (2)	0.050 (9)*	0.271 (12)
H21A	0.0273	0.6474	0.4649	0.060*	0.271 (12)
C22A	0.135 (5)	0.4023 (14)	0.487 (2)	0.042*	0.271 (12)
H22A	0.1609	0.3403	0.4942	0.050*	0.271 (12)
C23A	−0.024 (5)	0.444 (3)	0.553 (2)	0.037 (12)*	0.271 (12)
C24A	−0.071 (6)	0.535 (2)	0.548 (3)	0.071 (18)*	0.271 (12)
H24A	−0.1794	0.5619	0.5932	0.085*	0.271 (12)
O25A	−0.135 (10)	0.397 (6)	0.622 (5)	0.061 (13)*	0.271 (12)
H25A	−0.18 (5)	0.439 (15)	0.67 (3)	0.091*	0.271 (12)

### Atomic displacement parameters (Å^2^)

**Table:**

	*U*^11^	*U*^22^	*U*^33^	*U*^12^	*U*^13^	*U*^23^
O1	0.110 (3)	0.049 (2)	0.112 (4)	−0.013 (2)	0.075 (3)	0.007 (2)
C2	0.060 (4)	0.049 (3)	0.063 (4)	0.004 (3)	0.027 (3)	0.003 (3)
C3	0.057 (4)	0.034 (3)	0.048 (4)	0.000 (2)	0.018 (3)	0.007 (2)
C4	0.080 (4)	0.040 (3)	0.097 (5)	−0.001 (3)	0.040 (4)	0.002 (3)
C5	0.054 (4)	0.052 (4)	0.070 (4)	0.000 (3)	0.034 (3)	0.005 (3)
C6	0.063 (4)	0.035 (3)	0.069 (4)	0.009 (3)	0.039 (3)	0.002 (3)
C7	0.052 (3)	0.037 (3)	0.056 (4)	0.001 (3)	0.030 (3)	0.002 (2)
C8	0.070 (4)	0.035 (3)	0.079 (4)	−0.003 (3)	0.047 (3)	0.001 (3)
C9	0.059 (4)	0.038 (3)	0.076 (4)	0.004 (3)	0.043 (3)	0.001 (3)
N10	0.069 (3)	0.030 (2)	0.067 (3)	0.003 (2)	0.046 (3)	−0.001 (2)
C11	0.052 (3)	0.044 (3)	0.058 (4)	−0.003 (3)	0.021 (3)	0.001 (3)
O12	0.073 (3)	0.040 (2)	0.088 (3)	0.0043 (18)	0.054 (2)	0.0006 (19)
C13	0.061 (4)	0.037 (3)	0.063 (4)	−0.001 (3)	0.033 (3)	0.000 (2)
O14	0.074 (3)	0.040 (2)	0.098 (3)	−0.0014 (18)	0.058 (2)	0.0079 (19)
C15	0.044 (6)	0.046 (6)	0.038 (6)	−0.011 (4)	−0.004 (5)	0.017 (5)
O16	0.076 (6)	0.022 (3)	0.148 (10)	0.006 (3)	0.081 (6)	0.012 (3)
C17	0.046 (5)	0.045 (5)	0.053 (6)	−0.006 (4)	0.008 (4)	0.010 (4)
C18	0.070 (6)	0.076	0.067 (7)	−0.017 (5)	0.020 (5)	0.015 (5)
C19	0.054 (6)	0.090 (7)	0.044 (6)	−0.025 (5)	0.009 (4)	0.012 (5)
C20	0.063 (5)	0.064	0.083 (7)	−0.008 (5)	0.052 (5)	−0.002 (6)
C21	0.059 (6)	0.081 (7)	0.050 (7)	−0.016 (5)	0.012 (5)	0.014 (6)
C22	0.089 (7)	0.085	0.093 (8)	0.010 (7)	0.048 (6)	0.002 (7)
C23	0.056 (8)	0.088 (12)	0.057 (8)	−0.004 (6)	0.031 (5)	0.018 (6)
C24	0.051 (7)	0.071 (8)	0.066 (7)	−0.005 (5)	0.033 (5)	0.013 (6)
O25	0.085 (8)	0.064 (9)	0.108 (10)	−0.001 (5)	0.068 (6)	0.018 (6)

### Geometric parameters (Å, º)

**Table:**

O1—C2	1.226 (6)	C18—H18B	0.9700
C2—C3	1.471 (7)	C19—C21	1.352 (13)
C2—C4	1.512 (7)	C19—C20	1.353 (13)
C3—C9	1.389 (6)	C20—C22	1.391 (12)
C3—C5	1.398 (7)	C20—H20	0.9300
C4—H4A	0.9600	C21—C24	1.378 (13)
C4—H4B	0.9600	C21—H21	0.9300
C4—H4C	0.9600	C22—C23	1.391 (13)
C5—C6	1.372 (7)	C22—H22	0.9300
C5—H5	0.9300	C23—C24	1.35 (2)
C6—C7	1.380 (7)	C23—O25	1.46 (3)
C6—H6	0.9300	C24—H24	0.9300
C7—C8	1.381 (7)	O25—H25	0.95 (12)
C7—N10	1.411 (6)	C15A—O16A	1.24 (3)
C8—C9	1.377 (7)	C15A—C17A	1.53 (2)
C8—H8	0.9300	C17A—C18A	1.50 (4)
C9—H9	0.9300	C17A—H17C	0.9700
N10—C11	1.354 (6)	C17A—H17D	0.9700
N10—H10	0.86 (5)	C18A—C19A	1.51 (3)
C11—O12	1.212 (6)	C18A—H18C	0.9700
C11—C13	1.503 (7)	C18A—H18D	0.9700
C13—O14	1.421 (5)	C19A—C21A	1.37 (2)
C13—H13A	0.9700	C19A—C20A	1.41 (2)
C13—H13B	0.9700	C20A—C22A	1.37 (2)
O14—C15A	1.37 (3)	C20A—H20A	0.9300
O14—C15	1.373 (11)	C21A—C24A	1.39 (3)
C15—O16	1.176 (12)	C21A—H21A	0.9300
C15—C17	1.514 (10)	C22A—C23A	1.38 (2)
C17—C18	1.525 (13)	C22A—H22A	0.9300
C17—H17A	0.9700	C23A—O25A	1.26 (7)
C17—H17B	0.9700	C23A—C24A	1.37 (3)
C18—C19	1.525 (11)	C24A—H24A	0.9300
C18—H18A	0.9700	O25A—H25A	0.90 (3)
			
O1—C2—C3	120.8 (5)	H18A—C18—H18B	107.7
O1—C2—C4	119.3 (5)	C21—C19—C20	118.0 (8)
C3—C2—C4	119.9 (5)	C21—C19—C18	121.9 (9)
C9—C3—C5	117.6 (4)	C20—C19—C18	120.1 (9)
C9—C3—C2	123.7 (4)	C19—C20—C22	121.7 (8)
C5—C3—C2	118.7 (5)	C19—C20—H20	119.2
C2—C4—H4A	109.5	C22—C20—H20	119.2
C2—C4—H4B	109.5	C19—C21—C24	121.7 (12)
H4A—C4—H4B	109.5	C19—C21—H21	119.2
C2—C4—H4C	109.5	C24—C21—H21	119.2
H4A—C4—H4C	109.5	C20—C22—C23	119.2 (11)
H4B—C4—H4C	109.5	C20—C22—H22	120.4
C6—C5—C3	120.6 (5)	C23—C22—H22	120.4
C6—C5—H5	119.7	C24—C23—C22	118.5 (12)
C3—C5—H5	119.7	C24—C23—O25	126.0 (17)
C5—C6—C7	120.8 (5)	C22—C23—O25	115.6 (19)
C5—C6—H6	119.6	C23—C24—C21	121.0 (12)
C7—C6—H6	119.6	C23—C24—H24	119.5
C6—C7—C8	119.5 (4)	C21—C24—H24	119.5
C6—C7—N10	116.5 (4)	C23—O25—H25	105 (8)
C8—C7—N10	124.0 (4)	O16A—C15A—O14	113 (3)
C9—C8—C7	119.6 (5)	O16A—C15A—C17A	135 (3)
C9—C8—H8	120.2	O14—C15A—C17A	105.6 (19)
C7—C8—H8	120.2	C18A—C17A—C15A	108 (2)
C8—C9—C3	121.8 (5)	C18A—C17A—H17C	110.1
C8—C9—H9	119.1	C15A—C17A—H17C	110.1
C3—C9—H9	119.1	C18A—C17A—H17D	110.1
C11—N10—C7	130.0 (4)	C15A—C17A—H17D	110.1
C11—N10—H10	118 (4)	H17C—C17A—H17D	108.4
C7—N10—H10	112 (4)	C17A—C18A—C19A	113 (2)
O12—C11—N10	125.0 (5)	C17A—C18A—H18C	108.9
O12—C11—C13	123.6 (5)	C19A—C18A—H18C	108.9
N10—C11—C13	111.4 (4)	C17A—C18A—H18D	108.9
O14—C13—C11	109.0 (4)	C19A—C18A—H18D	108.9
O14—C13—H13A	109.9	H18C—C18A—H18D	107.7
C11—C13—H13A	109.9	C21A—C19A—C20A	119 (2)
O14—C13—H13B	109.9	C21A—C19A—C18A	128 (2)
C11—C13—H13B	109.9	C20A—C19A—C18A	112.3 (19)
H13A—C13—H13B	108.3	C22A—C20A—C19A	119 (2)
C15A—O14—C13	113.9 (10)	C22A—C20A—H20A	120.3
C15—O14—C13	114.3 (5)	C19A—C20A—H20A	120.3
O16—C15—O14	122.3 (8)	C19A—C21A—C24A	122 (2)
O16—C15—C17	123.7 (9)	C19A—C21A—H21A	118.8
O14—C15—C17	112.7 (8)	C24A—C21A—H21A	118.8
C15—C17—C18	110.2 (7)	C20A—C22A—C23A	119 (2)
C15—C17—H17A	109.6	C20A—C22A—H22A	120.5
C18—C17—H17A	109.6	C23A—C22A—H22A	120.5
C15—C17—H17B	109.6	O25A—C23A—C24A	117 (5)
C18—C17—H17B	109.6	O25A—C23A—C22A	119 (5)
H17A—C17—H17B	108.1	C24A—C23A—C22A	124 (3)
C19—C18—C17	113.5 (5)	C23A—C24A—C21A	116 (3)
C19—C18—H18A	108.9	C23A—C24A—H24A	122.0
C17—C18—H18A	108.9	C21A—C24A—H24A	122.0
C19—C18—H18B	108.9	C23A—O25A—H25A	103 (10)
C17—C18—H18B	108.9		
			
O1—C2—C3—C9	−179.6 (5)	C17—C18—C19—C20	88.3 (8)
C4—C2—C3—C9	0.7 (8)	C21—C19—C20—C22	−0.5 (5)
O1—C2—C3—C5	−0.9 (9)	C18—C19—C20—C22	179.7 (3)
C4—C2—C3—C5	179.3 (5)	C20—C19—C21—C24	0.5 (8)
C9—C3—C5—C6	0.5 (8)	C18—C19—C21—C24	−179.7 (5)
C2—C3—C5—C6	−178.2 (5)	C19—C20—C22—C23	0.6 (7)
C3—C5—C6—C7	0.9 (8)	C20—C22—C23—C24	−0.6 (9)
C5—C6—C7—C8	−1.5 (9)	C20—C22—C23—O25	179.7 (5)
C5—C6—C7—N10	179.1 (5)	C22—C23—C24—C21	0.6 (10)
C6—C7—C8—C9	0.7 (9)	O25—C23—C24—C21	−179.7 (6)
N10—C7—C8—C9	180.0 (5)	C19—C21—C24—C23	−0.6 (10)
C7—C8—C9—C3	0.8 (9)	C15—O14—C15A—O16A	60 (4)
C5—C3—C9—C8	−1.3 (8)	C13—O14—C15A—O16A	−36 (4)
C2—C3—C9—C8	177.3 (6)	C15—O14—C15A—C17A	−96 (3)
C6—C7—N10—C11	−179.6 (5)	C13—O14—C15A—C17A	167.6 (15)
C8—C7—N10—C11	1.0 (10)	O16A—C15A—C17A—C18A	14 (7)
C7—N10—C11—O12	2.6 (10)	O14—C15A—C17A—C18A	162 (2)
C7—N10—C11—C13	−176.3 (5)	C15A—C17A—C18A—C19A	173.0 (16)
O12—C11—C13—O14	2.7 (7)	C17A—C18A—C19A—C21A	6 (2)
N10—C11—C13—O14	−178.4 (5)	C17A—C18A—C19A—C20A	−174 (2)
C11—C13—O14—C15A	−158.1 (12)	C21A—C19A—C20A—C22A	−0.1 (5)
C11—C13—O14—C15	175.5 (7)	C18A—C19A—C20A—C22A	179.9 (4)
C15A—O14—C15—O16	−85 (3)	C20A—C19A—C21A—C24A	0.0 (8)
C13—O14—C15—O16	9.0 (15)	C18A—C19A—C21A—C24A	−180.0 (5)
C15A—O14—C15—C17	82 (3)	C19A—C20A—C22A—C23A	0.1 (8)
C13—O14—C15—C17	176.8 (6)	C20A—C22A—C23A—O25A	179.9 (6)
O16—C15—C17—C18	80.5 (15)	C20A—C22A—C23A—C24A	−0.1 (11)
O14—C15—C17—C18	−87.2 (10)	O25A—C23A—C24A—C21A	−179.9 (7)
C15—C17—C18—C19	−170.4 (8)	C22A—C23A—C24A—C21A	0.1 (11)
C17—C18—C19—C21	−91.5 (8)	C19A—C21A—C24A—C23A	−0.1 (10)

### Hydrogen-bond geometry (Å, º)

**Table:**

*D*—H···*A*	*D*—H	H···*A*	*D*···*A*	*D*—H···*A*
N10—H10···O1^i^	0.86 (5)	2.11 (5)	2.925 (5)	159 (4)
O25—H25···O12^ii^	0.95 (12)	1.93 (11)	2.87 (3)	172 (13)
C4—H4*A*···O16^iii^	0.96	2.59	3.416 (10)	144
C13—H13*B*···O25^iv^	0.97	2.60	3.458 (17)	147
C13—H13*B*···O25*A*^iv^	0.97	2.50	3.34 (4)	145
C24—H24···O12^ii^	0.93	2.57	3.284 (11)	133

Symmetry codes: (i) −*x*+2, *y*−1/2, −*z*; (ii) −*x*, *y*−1/2, −*z*+1; (iii) *x*, *y*+1, *z*; (iv) −*x*+1, *y*+1/2, −*z*+1.
